# TMEM106B modulates disease severity in genetic frontotemporal dementia phenoconverters

**DOI:** 10.1002/alz70856_103532

**Published:** 2025-12-25

**Authors:** Maurice Pasternak, Saira S. Mirza, Andrew D. Paterson, Carmela Tartaglia, Sara Mitchell, Sandra E. Black, Morris Freedman, David F. Tang‐Wai, Ekaterina Rogaeva, David M Cash, Martina Bocchetta, John van Swieten, Robert Laforce, Fabrizio Tagliavini, Barbara Borroni, Daniela Galimberti, James B Rowe, Caroline Graff, Elizabeth Finger, Sandro Sorbi, Alexandre de Mendonça, Christopher Butler, Alexander Gerhard, Raquel Sánchez‐Valle, Fermin Moreno, Matthis Synofzik, Rik Vandenberghe, Simon Ducharme, Johannes Levin, Markus Otto, Isabel Santana, Jonathan D. Rohrer, Mario Masellis, GENFI

**Affiliations:** ^1^ Hurvitz Brain Sciences Program, Sunnybrook Research Institute, Toronto, ON, Canada; ^2^ The Hospital for Sick Children, Toronto, ON, Canada; ^3^ University of Toronto, Toronto, ON, Canada; ^4^ Memory Clinic, Toronto Western Hospital, University Health Network, Toronto, ON, Canada; ^5^ University of Toronto Department of Medicine, Toronto, ON, Canada; ^6^ Division of Neurology, Department of Medicine, University of Toronto, Toronto, ON, Canada; ^7^ Hurvitz Brain Sciences Program, Toronto, ON, Canada; ^8^ Dr. Sandra E. Black Centre for Brain Resilience and Recovery, LC Campbell Cognitive Neurology, Hurvitz Brain Sciences Program, Sunnybrook Research Institute, University of Toronto, Toronto, ON, Canada; ^9^ Sunnybrook Research Institute, University of Toronto, Toronto, ON, Canada; ^10^ Rotman Research Institute, Baycrest Health Sciences, Toronto, ON, Canada; ^11^ Department of Medicine, University of Toronto, Toronto, ON, Canada; ^12^ Krembil Brain Institute, University Health Network (UHN), Toronto, ON, Canada; ^13^ Rotman Research Institute at Baycrest, Toronto, ON, Canada; ^14^ Tanz Centre for Research in Neurodegenerative Diseases, University of Toronto, Toronto, ON, Canada; ^15^ Dementia Research Centre, UCL Queen Square Institute of Neurology, University College London, London, United Kingdom; ^16^ Dementia Research Centre, UCL Queen Square Institute of Neurology, University College London, London, London, United Kingdom; ^17^ Department of Neurology, Erasmus Medical Center, Rotterdam, Netherlands; ^18^ Clinique Interdisciplinaire de mémoire, CHU de Québec ‐ Université Laval, Quebec City, QC, Canada; ^19^ Fondazione Istituto di Ricovero e Cura a Carattere Scientifico, Istituto Neurologica Carlo Besta, Milan, ‐, Italy; ^20^ Department of Clinical and Experimental Sciences, University of Brescia, Brescia, Italy; ^21^ Fondazione IRCCS Ca’ Granda, Ospedale Policlinico, Neurodegenerative Diseases Unit, Milan, Italy; ^22^ Department of Biomedical, Surgical and Dental Sciences, University of Milan, Milan, Italy; ^23^ Cambridge University Hospitals NHS Foundation Trust, Cambridge, United Kingdom; ^24^ Department of Clinical Neurosciences, University of Cambridge, Cambridge, Cambridgeshire, United Kingdom; ^25^ Medical Research Council Cognition and Brain Sciences Unit, Cambridge, United Kingdom; ^26^ Department of Neurobiology, Care Sciences and Society, Center for Alzheimer Research, Division of Neurogeriatrics, Karolinska Institutet, Stockholm, ‐, Sweden; ^27^ Unit for Hereditary Dementia, Theme Inflammation and Aging, Karolinska University Hospital‐Solna, Stockholm, Stockholm, Sweden; ^28^ University of Western Ontario, London, ON, Canada; ^29^ IRCCS Fondazione Don Carlo Gnocchi, Florence, Florence, Italy; ^30^ University of Florence, Florence, Florence, Italy; ^31^ Faculty of Medicine, University of Lisbon, Lisbon, Portugal; ^32^ University of Oxford, Oxford, United Kingdom; ^33^ Department of Brain Sciences, Imperial College London, London, United Kingdom; ^34^ Division of Neuroscience and Experimental Psychology, Wolfson Molecular Imaging Centre, University of Manchester, Manchester, ‐, United Kingdom; ^35^ Department of Geriatric Medicine, Klinikum Hochsauerland, Arnsberg, Arnsberg, Germany; ^36^ Alzheimer's disease and other cognitive disorders Unit. Hospital Clínic de Barcelona; FRCB‐IDIBAPS; University of Barcelona, Barcelona, Spain; ^37^ Hospital Universitario Donostia, San Sebastian, Gipuzkoa, Spain; ^38^ Biodonostia Health Research Institute, San Sebastian, Gipuzkoa, Spain; ^39^ Department of Neurodegenerative Diseases, Hertie‐Institute for Clinical Brain Research and Center of Neurology, University of Tübingen, Tübingen, Germany; ^40^ German Center for Neurodegenerative Diseases (DZNE), University of Tubingen, Tubingen, Germany; ^41^ UZ Leuven, Leuven, Belgium; ^42^ Leuven Brain Institute, Leuven, Belgium; ^43^ Laboratory for Parkinson Research, Leuven Brain Institute, KU Leuven, Leuven, Belgium; ^44^ Douglas Mental Health University Institute, Montreal, QC, Canada; ^45^ McConnell Brain Imaging Centre, Montreal Neurological Institute, McGill University, Montreal, QC, Canada; ^46^ Munich Cluster for Systems Neurology (SyNergy), Munich, Munich, Germany; ^47^ Neurologische Klinik und Poliklinik, Ludwig‐Maximilians‐Universität, Munich; German Center for Neurodegenerative Diseases (DZNE), Munich; Munich Cluster of Systems Neurology, Munich, Germany; ^48^ German Center for Neurodegenerative Diseases (DZNE), Munich, Bavaria, Germany; ^49^ University Hospital Ulm, Ulm, ‐, Germany; ^50^ Center for Neuroscience and Cell Biology, Faculty of Medicine, University of Coimbra, Coimbra, Coimbra, Portugal; ^51^ Neurology Service, Faculty of Medicine, University Hospital of Coimbra (HUC), University of Coimbra, Coimbra, Coimbra, Portugal; ^52^ Dementia Research Centre, Queen Square Institute of Neurology, University College London, London, ‐, United Kingdom; ^53^ Division of Neurology, Department of Medicine, Sunnybrook Health Sciences Centre, Toronto, ON, Canada; ^54^ Cognitive and Movement Disorders Clinic, Sunnybrook Health Sciences Center, Toronto, ON, Canada

## Abstract

**Background:**

A common variant within TMEM106B is associated with risk for Frontotemporal Lobar Degeneration‐Tar DNA binding Protein‐43 (FTLD‐TDP). A recent study has shown that the minor allele G of TMEM106B‐rs1990622 confers protection against FTLD‐TDP in symptomatic mutation carriers through reductions in NfL serum levels, brain atrophy, and cognitive decline. It is unknown whether this protective effect is present in phenoconverters of the disease.

**Method:**

We included 518 participants from the GENetic Frontotemporal dementia Initiative (GENFI), which recruits genetic FTD cases and their family members, both carriers and non‐carriers of FTD mutations. Of these, 21 were phenoconverters, 209 were non‐carrier controls, 70 were presymptomatic and 45 symptomatic C9orf72 carriers, 92 presymptomatic and 29 symptomatic GRN carriers, and 39 presymptomatic and 13 symptomatic MAPT carriers. Effects of interaction between TMEM106B‐rs1990622 and phenoconverter status were examined using mixed effects models, with a random effects structure featuring subjects nested within families and fixed effects for age at baseline and sex. Serum neurofilament light chain (NfL) was measured using the Simoa platform. Cognitive assessment included the Mini‐Mental State Examination (MMSE), tests of attention, processing speed, executive function, and language, as well as the Cambridge Behavioural Inventory (CBI), with mixed effects also including years of education as a covariate. Brain volumetry was assessed using T1‐weighted MRI and these mixed effect models also included additional covariates of total intracranial volume and scanner site.

**Result:**

In phenoconverters, each copy of the protective allele G was associated with a significant reduction in the rate of serum NfL accumulation (‐5.33 pg/mL/year; *p* =  7.79 × 10^−9^). Structural imaging analyses revealed decreased rates of atrophy in fronto‐orbital regions and the insular cortex among protective allele carriers. Cognitive trajectories showed significantly slower decline across multiple domains including general cognition (MMSE; *p* =  0.003), attention and processing speed (*p* = 2.2 × 10^−4^), executive function (*p* = 2.6 × 10^−7^), language (*p* = 2.9 × 10^−3^), and behavioural symptoms as measured by CBI (*p* = 9.5 × 10^−3^).

**Conclusion:**

The TMEM106B‐rs1990622 protective variant significantly modulates disease progression in genetic FTD phenoconverters across multiple markers, suggesting its potential as a therapeutic target.

